# The “Homœopathic” Medical Society of Penna.

**Published:** 1888-12

**Authors:** 


### THE "HOMŒOPATHIC" MEDICAL SOCIETY OF PENNSYLVANIA.

The last meeting of this Society was held in this city; a few homœopathic papers were read. The greater number of the papers were out-and-out eclectic and of a poor quality. We quote a few samples; our readers will remember that these physicians are mostly *professors* and to them is intrusted the teaching of future "homœopathic" physicians.

G. Maxwell Christine, M. D., read a paper on "The Leech and Cup as Aids in the Treatment of Disease." Dr. Christine argued against the rejection of the practice of blood-letting as a blind and prejudiced action.

The first paper on surgical topics presented was a valuable one on carbuncle by Dr. W. B. Van Lennep, of Philadelphia, entitled, "The Antiseptic Treatment of Carbuncle, Furuncle, and Kindred Affections." He invariably uses the remedies Arsenic, Carbo vegetabilis, Rhus toxicodendron, Hepar sulphur, and Carbolic acid injections, and Iodoform dressings locally.

Dr. Willard corresponded with Dr. Van Lennep in the latter's treatment of carbuncle with Carbolic acid, by dipping a bodkin in Carbolic acid, with which he punctured the wound. Dr. Thomas, who spoke next, advocated the same method of treatment, and gave several instances of its success in patients of his own. In cases not so far advanced he recommended treatment with caustic Potash. In all carbuncles the parts should not only be thoroughly cleansed, but antiseptically treated.

John E. James, M. D., Professor of Surgery in the Hahnemann College, succeeded Dr. Thomas. He cited cases in which, in a disease similar to carbuncle, a hypodermic injection of Carbolic acid had been made under the diseased surface, with the result of drying up the decayed matter. For the relief of the prostration that accompanies suppurating diseases, from one-half to two or three spoonfuls of the tincture of Peruvian bark.

The discussion was brought to a close by Dr. Joseph E. Jones, of West Chester, who said that in the next case of bone felon that comes under his notice he will use the hypodermic syringe with a solution of Carbolic acid, as superior in all respects to any other method.

Dr. Mohr read a paper giving the history of an interesting and remarkable case of malignant growth of cancer cured by accident. The patient, a woman, was being treated with Arsenic. Erysipelas broke out and cured the cancer. This accidental cure, Dr. Mohr declared, has possibly opened up a way by which cancer can be cured with the virus of erysipelas. The paper created much talk, and Dr. Mohr was asked a great many questions regarding his patient, who is now in better flesh than she has been for six years.

Dr. Mohr's report illustrates the haste physicians make to form *theories;* presuming upon this case, Dr. Mohr thinks the virus of erysipelas may prove a specific for cancer!

Such proceedings as those of this Society bear ample fruit in educating young men in empiricism and in bringing contempt upon Homœopathy. At the last meeting of the "New York Academy of Medicine," a Dr. McIntire read a suggestive paper, in which he declared that the allopaths and the homœopaths practiced alike; that they differed *only in name.* His paper was entitled, "Which is the Liberal School?" and was suggested by some correspondence with a graduate of a Hahnemann college, who asserted that whenever it was *necessary* the graduates of these schools went beyond the theory of therapeutics indicated by their name and, consequently, those who refused to consult with them were illiberal. He quoted from the address of Dr. Pitcairn before the last annual meeting of the Homœopathic Medical Society of Pennsylvania to substantiate the statement of his correspondent that Homœopathy did not mean exclusively practicing by the law of similars. If so, then there is no essential difference in the method of treatment of either "school," and that body of physicians who make a class of themselves by calling themselves by any exclusive name become thereby the illiberal school. If they drop this name, they will find no barrier in their method of treating disease from the most cordial intercourse with any or all physicians.

